# Medical Opinion and Movement

**Published:** 1906-07-14

**Authors:** 


### Medical Opinion and Movement.

The election of Fellows to the Council of the Royal College of Surgeons produced an unusually heavy poll. The provincial Fellows came forward in great strength, and elected Mr. George A. Wright, of Manchester, at the head of the poll. Mr. Henry Morris, of Middlesex Hospital, came next, and then Mr. F. Richardson Cross, of Bristol, both of whom were accordingly re-elected. We hope next year to see Mr. Charters J. Symonds at the head of the poll.

Some agitation is on foot to move the authorities by enforcing the law to put a stop to the obnoxious smells which are at present emitted from the scores of evil-smelling factories between Stratford and Bow. We should imagine that an evil of this kind is one which intimately concerns the Great Eastern Railway authorities, and they may find it necessary in self-defence to combine with the local authorities, and so secure the abatement of an evil which may tend to render many a business man travelling by this route unfit for his work on arrival in the city.

The Bradford Corporation have adopted certain therapeutic measures at the Bradford Baths which have been condemned by the medical profession in that city. On the motion of Dr. Hime it was resolved to be highly undesirable that the Bradford Council should carry on the treatment of disease for profit through officials of the Corporation Baths, who are not legally qualified medical practitioners, who treat diseases which are not of an epidemic character, and that such practices are illegal. We imagine that the deputation of representatives of the profession who are to wait upon the Bradford Corporation will have little difficulty in convincing the Council that they have not authority to carry out any such treatment, and that the practice will be discontinued, or that any treatment provided will be placed under the direct control and administration of qualified practitioners.

Messrs. Armour and Co., who are issuing a number of circulars on the subject, have sent us a document entitled "Notes on the *Lancet* articles and 'The Jungle,' Extract from the Report of the Committee of the U.S. Department of Agriculture, dated April 13th last," with a request that we will call attention to it. In acceding to this request we are bound to say that we feel all impartial persons who study this document will agree that it elaborately confirms and strengthens the criticisms contained in the *Lancet* articles. If the report of this committee represents the best defence that the U.S. Department of Agriculture can make of the existing condition of the packing establishments in Chicago, President Roosevelt is justified, and more than justified in our opinion, in the course he has taken, and it is essential that everything the President has contended for shall be enacted in its entirety by the Legislature.

The action of the Select Committee of the House of Lords, presided over by Lord Camperdown, which has refused to grant the powers sought by the London County Council to establish a street ambulance system in London, is as unaccountable as it is likely to prove unpopular. We can only suppose that the Lords object to the scheme owing to its limited and experimental character, which they may have deemed to be inadequate. If this be so, Lord Camperdown and his Committee should lose no time in making the facts clear, for otherwise they are likely to incur an amount of odium which it would be wise to avoid. The London County Council ought to have the power to establish and maintain an ambulance service for dealing with all cases of accident or illness in the streets or other public places in the county of London. The matter is so important that we hope something may be done even yet to restore the omitted clauses to the Bill now before Parliament. Sir William Collins has borne the heat and burden of the recent fight for an ambulance system for London, and all Londoners owe him a debt of gratitude in consequence which we hope will be fully recognised at the proper time.

The seventy-fourth annual meeting of the British Medical Association at Toronto, in Canada, next month promises to be rich in scientific interest. There will be 13 sections, in each of which points of the highest importance will be brought forward and discussed by some of the most eminent practitioners in this country. The section of medicine will be presided over by Sir Thomas Barlow, and the subject of blood-pressure in relation to disease will be discussed by such authorities as Dr. Dawson (Baltimore), Dr. G. A. Gibson (Edinburgh), Sir William Broadbent (London), who deals with the "Clinical Significance and Therapeutic Indications of Variations in Blood-pressure"; Professor Clifford Albutt (Cambridge), "Blood-pressure in Arterial Sclerosis"; Dr. Mackenzie (Burnley), Professor Osler (Oxford), and Dr. Rose Bradford (London). An important discussion on over- and under-nutrition, with special reference to proteid metabolism, is sure to attract a large house of physicians and physiologists. We note with pleasure that Professor Adami is to preside over the important section of pathology, and will also read a paper on fluid crystals and their relationship to arterio-sclerosis and other pathological conditions. The discussion on the pathogenic protozoa should prove a new feature to many practitioners who have not had the leisure to follow the recent discoveries in this region. In the section of physiology a large number of prominent American, as well as French and German, physiologists have accepted invitations to attend, under the presidency of Dr. Halliburton (London). In the section of State medicine Dr. Cooper Pattin, of Norwich, will deal with "The Training and Supervision of Midwives" and "The Control of the Milk Supply"; and such subjects as the Notification of Phthisis, Isolation Hospitals, Inspection of School Children will form part of a full programme. Sir Hector Clare Cameron (Glasgow) will preside over the surgery section, and Dr. Donald MacAlister over that of Therapeutics. The special subjects are the "Pharmacology and Therapeutics of the Kidney" and "Serum Therapy and the perennial discussion on the Therapeutic Value of Alcohol." In the special sections Dr. Dundas-Grant (London) will preside over Laryngology, and Mr. Marcus Gunn (London) over Ophthalmology.

